# Prostatic resection cavity large stone post transurethral resection of the prostate (TURP). A rare case scenario

**DOI:** 10.1016/j.ijscr.2021.105726

**Published:** 2021-03-06

**Authors:** Diaa-Eldin Taha

**Affiliations:** Urology Department, Faculty of Medicine, KafrElsheikh University, Kafrelsheikh, Egypt

**Keywords:** Prostatic cavity stone, Post TURP, Posterior urethra, Bladder outlet obstruction (BOO)

## Abstract

**Introduction:**

Transurethral resection of the prostate (TURP) compromise the mainstay surgical treatment of LUTS due to benign prostatic hyperplasia (BPH). The storage symptoms post TURP may be attributed to urinary tract infection (UTI), preoperative detrusor over-activities, and residual prostatic adenoma causing voiding symptoms.

**Presentation of case:**

A 56 year old male presented storage LUTS (mainly frequency and urgency) since two years. Two years earlier, he underwent uncomplicated monopolar TURP. The patient has occasional straining and intermittent urine. No history of hematuria. No notable medical history was present. Digital rectal examination showed small prostate. Anal tone and Bulbocavernosal reflex were intact. CT showed a large vesical stone extending into the prostatic fossa measuring 51.5 mm × 67.0 mm. The patient was managed by suprapubic cystolitholapaxy.

**Discussion:**

Post TURP LUTS necessitates evaluation with a thorough history and physical, including International Prostate Symptom Score, and urine culture to rule out infection. In a rare case report, delayed occurrence of storage and obstructive voiding symptoms after TURP can be caused by dystrophic calcification of the prostatic resection cavity. The stone could have been due to a metal or plastic piece of the resectoscope embedded in the prostatic cavity, but, this postulation was deferred based on the non-attached stone to the mucosa as confirmed by cystoscope. In such case, based on the large stone burden, more cost would be a potential burden, and longer operative time, the open cystolitholapaxy is the modality of choice.

**Conclusion:**

Prostatic cavity stone is a rare pathology. Incidental stone occupying the prostatic fossa post TURP is a remote possibility but it should by highlighted to raise urologist awareness for its possibility.

## Highlights

• Prostatic cavity stone post TURP should be highlighted in the surgeon mind in patient with persistent storage LUTS post TURP.
• CT adequately burden the stone suspected.
• Persistent storage symptoms post TURP was the main complaint.
• Incidental stone occupying the prostatic fossa post TURP is a remote possibility but it should by highlighted to raise urologist awareness for its possibility.

## 1 Introduction

Transurethral resection of the prostate (TURP) is the gold standard surgical therapy for lower urinary tract symptoms (LUTS) owing to senile prostatic enlargement. Following TURP, LUTS may persist in a percentage of patients. Persistent LUTS necessitates proper evaluation and management [1].

In a rare case report, delayed occurrence of storage and obstructive voiding symptoms after TURP can be caused by dystrophic calcification of the prostatic resection cavity and might be misinterpreted as post-TURP infection. The mechanism of dystrophic calcification entails minimizing tissue trauma by cautious removal of calcifications rather than performing extensive Re-TURP [2].

In our case, a rare presentation of storage LUTS as result of prostatic cavity stone extending into the bladder with same continuum, elicite the core issue of post TURP storage LUTS.

## 2 Case presentation

A 56 year old male presented to our clinic, complaining of storage LUTS (mainly frequency and urgency) since two years. Two years earlier, he underwent uncomplicated monopolar TURP for LUTS owing to benign prostatic hyperplasia. The pathology of resected prostatic chips showed no inflammatory changes. The patient has occasional straining and intermittent urine. No history of hematuria. The patient was not smoker. No relevant drug history, family history including any relevant genetic information, and psychosocial history.

**Fig. 1:**
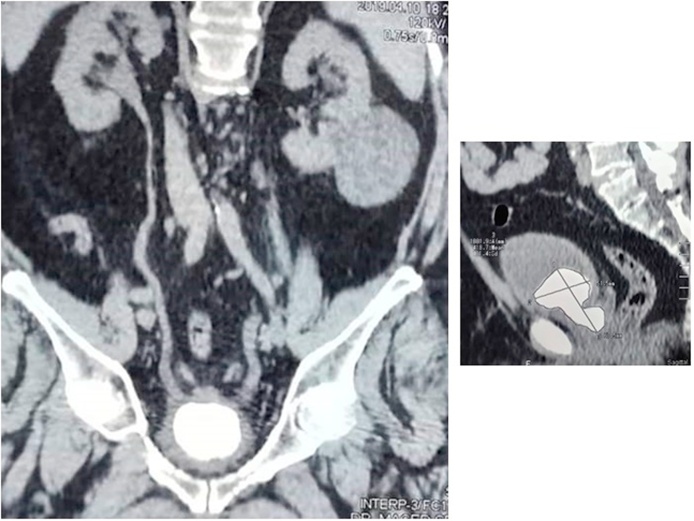
CT image of the prostatic cavity large stone measuring 3.2 × 2.8 cm.

Upon examination, the patient's abdomen was soft and lax, without palpable masses. Digital rectal examination showed small prostate. Anal tone and Bulbocavernosal reflex were intact. His laboratory blood tests, urinalysis and culture were normal. Ultrasonography of the urinary tract showed normal kidneys, but the urinary bladder showed a 3.2 × 2.8 cm vesical stone. CT showed a large vesical stone extending into the prostatic fossa measuring 51.5 mm × 67.0 mm (Fig. 1).

The patient was counseled about the possible surgical management and the different complications of each. Written informed consent was obtained from the patient. Preoperative urine culture was negative for organisms.

**Fig. 2:**
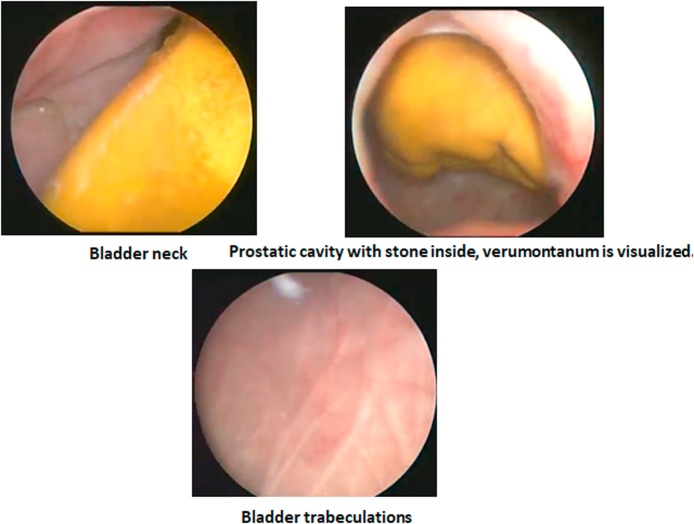
Cystoscopic view of the prostatic cavity with the occupied stone inside. Mild bladder trabeculations.

The author who is a consultant and lecturer of urology in a tertiary healthcare center, by the aid of a 17 F cystoscope, showed a normal distal urethra. Just after passing the verumontanum, the stone become visible inside the prostatic fossa with intact rhabdosphincter (Fig. 2). The fossa is occupied by the stone, despite the scope can pass near the stone to enter the bladder with visualization of the bladder neck (Fig. 2). The bladder mucosa showed mild trabeculation without masses or stones (Fig. 2).

**Fig. 3:**
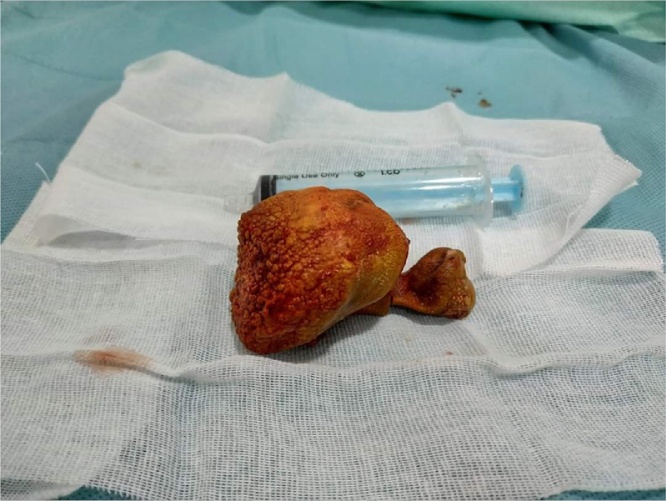
Extracted prostatic cavity stone.

The patient was repositioned for suprapubic cystolitholapaxy. Midline suprpubic incision with dissection of the bladder experitoneally helped extraction of the stone (Fig. 3). Eventually, closure of the wound in layers.

Two weeks later, the patient has no urgency, frequency, and the urine flow was adequate with 15 ml post void residual on pelvic US. After 3 months, the patient is doing fine with no storage or voiding symptoms. Stone analysis showed mixed crystal materials.

## 3 Discussion

TURP provides adequate treatment for storage and voiding symptoms [3]. The chronically obstructed bladder has been shown to vary in its expression of collagen, tissue factors, and receptors when compared to the normal bladder which could contribute to the pathophysiology of LUTS after TURP. Although androgen receptors exist in the urinary epithelium, the role of sex hormones in LUTS remains obscure [1].

Post TURP LUTS necessitates evaluation with a thorough history and physical, including International Prostate Symptom Score, and urine culture to rule out infection. Moreover, Noninvasive uroflowmetry, post-void residuals, and subsequent urodynamic study or cystoscopy should be utilized whenever needed [1].

In a rare case report, delayed occurrence of storage and obstructive voiding symptoms after TURP can be caused by dystrophic calcification of the prostatic resection cavity and might be misinterpreted as post-TURP infection. The mechanism of dystrophic calcification entails minimizing tissue trauma by cautious removal of calcifications rather than performing extensive Re-TURP [2].

In our case, a rare presentation of storage LUTS as result of prostatic cavity stone extending into the bladder with same continuum, elicite the core issue of post TURP storage LUTS. we offer the plausibility of clinical presentation and management of prostatic cavity stone post TURP.

The storage symptoms post TURP may be attributed to urinary tract infection (UTI) [4]. As there are significant relationship between bacteriuria and patient old age, past history of diabetes mellitus, large prostatic size, and positive preoperative urine analysis and culture [5]. We should control these risk factors, Moreover, adequately eradicating UTI will help improve patient symptoms.

The storage symptoms do not clearly correlate with bladder outlet obstruction (BOO), and may also occur independently of BOO. For this reason, OAB symptoms may persist after pharmacological and surgical treatment of BPH [6,7].

The stone could have been due to a metal or plastic piece of the resectoscope embedded in the prostatic cavity, but, this postulation was deferred based on the non-attached stone to the mucosa as confirmed by cystoscope.

In such case, based on the large stone burden, more cost would be a potential burden, and longer operative time, the open cystolitholapaxy is the modality of choice.

To the best of our knowledge, this is the first report for prostatic cavity stone post TURP that has a unique clinical picture.

## 4 Conclusion

Post TURP cavity stone is a rare scenario. Prostatic cavity stone post TURP should be investigated well in patient with persistent storage LUTS. CT adequately burden the stone suspected. The storage symptoms post TURP was the main complaint. Incidental stone occupying the prostatic fossa post TURP is a remote possibility but it should by highlighted to raise urologist awareness for its possibility.

## Declaration of Competing Interest

None.

## Funding

None.

## Ethical approval

Approval of the Institutional Review Board according to publish this case report was obtained. This case report manuscript follows the surgical case report (SCARE) Guidelines [8].

## Consent

Taken from the patient.

## Author contribution

Single author.

## Registration of research studies

Not Applicable.

## Guarantor

Single author.

## Provenance and peer review

Not commissioned, externally peer-reviewed.
